# Plasma proteins facilitates placental transfer of polystyrene particles

**DOI:** 10.1186/s12951-020-00676-5

**Published:** 2020-09-09

**Authors:** Michael M. Gruber, Birgit Hirschmugl, Natascha Berger, Magdalena Holter, Snježana Radulović, Gerd Leitinger, Laura Liesinger, Andrea Berghold, Eva Roblegg, Ruth Birner-Gruenberger, Vesna Bjelic-Radisic, Christian Wadsack

**Affiliations:** 1 Department of Obstetrics and Gynecology, Medical University of Graz, Auenbruggerplatz 14, 8036 Graz, Austria; 2 BioTechMed-Graz, Mozartgasse 12/II, 8010 Graz, Austria; 3 Institute for Medical Informatics, Statistics and Documentation, Medical University of Graz, Auenbruggerplatz 2, 8036 Graz, Austria; 4 Division of Molecular Biology and Biochemistry, Gottfried Schatz Research Center, Medical University of Graz, Neue Stiftingtalstraße 6/VI, 8010 Graz, Austria; 5 Research Unit Electron Microscopic Techniques, Division of Cell Biology, Histology and Embryology, Gottfried Schatz Research Center, Medical University of Graz, Neue Stiftingtalstraße 6/II, 8010 Graz, Austria; 6 Diagnostic and Research Institute of Pathology, Diagnostic and Research Center for Molecular BioMedicine, Medical University of Graz, Stiftingtalstrasse 6, 8010 Graz, Austria; 7 Omics Center Graz, BioTechMed-Graz, Stiftingtalstrasse 24, 8010 Graz, Austria; 8 Institute of Pharmaceutical Sciences, Department of Pharmaceutical Technology and Biopharmacy, University of Graz, Universitätsplatz 1/EG, 8010 Graz, Austria; 9 Institute of Chemical Technologies and Analytics, Faculty of Technical Chemistry, Vienna University of Technology-TU Wien, Getreidemarkt 9/164, 1060 Vienna, Austria

**Keywords:** Nanoparticle, Polystyrene, Biocorona, Dual ex vivo placental perfusion, Human placenta, Plasma proteins, Transfer

## Abstract

**Background:**

Nanoparticles, which are exposed to biological fluids are rapidly interacting with proteins and other biomolecules forming a corona. In addition to dimension, charge and material the distinct protein corona influences the interplay of nanoparticles with tissue barriers. In this study we were focused on the impact of in situ formed human plasma protein corona on the transfer of 80 nm polystyrene nanoparticles (PS-particles) across the human placenta. To study materno-to fetal PS transfer we used the human ex vivo placental perfusion approach, which represents an intact and physiological tissue barrier. To analyze the protein corona of PS particles we performed shotgun proteomics of isolated nanoparticles before and after tissue exposure.

**Results:**

Human plasma incubated with PS-particles of 80 nm and subsequent formed protein corona enhanced the transfer across the human placenta compared to PS-corona formed by bovine serum albumin and dextran which served as a control. Quantitative and qualitative changes of plasma proteins determined the changes in PS transfer across the barrier. Based on the analysis of the PS-proteome two candidate proteins, namely human albumin and immunoglobulin G were tested if these proteins may account for the enhanced PS-transfer across the placenta. Interestingly, the protein corona formed by human albumin significantly induced the transfer of PS-particles across the tissue compared to the formed IgG-corona.

**Conclusion:**

In total we demonstrate the PS corona dynamically and significantly evolves upon crossing the human placenta. Thus, the initial composition of PS particles in the maternal circulation is not predictive for their transfer characteristics and performance once beyond the barrier of the placenta. The precise mechanism of these effects remains to be elucidated but highlights the importance of using well designed biological models when testing nanoparticles for biomedical applications.
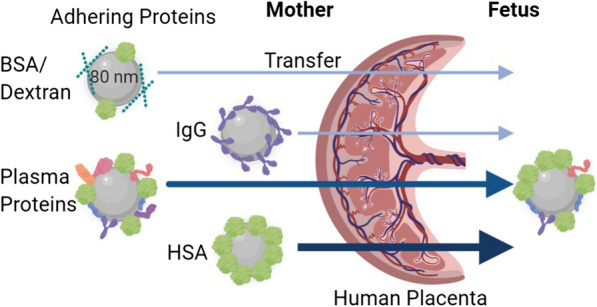

## Background

In the presence of biological fluids nanoparticle (NPs) are quickly coated with various biomolecules which ultimately form a biocorona [1]. The NP-biocorona is composed of proteins, lipids [2], nucleic acids [3] and metabolites [4]. The protein corona plays a pivotal role in the interaction of NPs with biological systems and has therefore been studied extensively [2, 5]. The composition of the protein corona depends on material properties, size, functionalization sites of the NP, as well as the interacting biological matrix [6–9]. The transport of NPs in the blood is influenced by many variables, including hydrodynamic flow forces [10, 11]. Immediate binding of NPs to plasma proteins is one of the most influential factors [12]. Thus, the NP-protein biocorona highly defines distribution and retention of NPs in tissues, as well as their interstitial transport which ultimately determines NP toxicity [12–14]. For such nano-interface in vitro studies cell culture media supplemented either with albumin, serum or anti-coagulated human plasma are frequently used [15]. These studies demonstrated supplementation specific effects of adhering proteins on NP retention at cellular targets [16, 17]. In addition, animal models have been developed to study more specifically the physiological role of the biocorona on NPs in systemic circulations [18]. Nevertheless, the composition of the protein corona is species-specific, which is an inherent limitation of animal models when extrapolating data to humans [19].

As an alternative to animal models cell-based spheroids, organ-on-a-chip approaches, and ex vivo human tissue settings have been developed [14, 18, 20–26]. One of these approaches is the ex vivo placenta perfusion model which has technical and physiological advantages compared to in vitro approaches and is considered as the golden standard for maternal to fetal transfer studies [27]. The given intact barriers of the perfused organ which is an absolute need for transfer studies and the translation of results to possible adverse effects on unborn life are strengths of this model. Therefore this approach has already been used to investigate NP-transfer across the placenta in different settings [28–30].

Our idea in this study was to extend the setting of this approach by investigating the effect of plasma proteins on the transfer of NPs. To the best of our knowledge data on such transfer studies in the human placenta is nonexistent. So far only cell lines were used to investigate the effect of protein corona on polystyrene nanoparticles (PS-particles) in presence of FBS [31]. Another study looked at the effect of FBS on cellular stress responses and the formation of protein NP aggregates in a first trimester cell line [32].

The present study aimed to examine the impact of a de novo forming protein corona on the transport of PS-particles across the human placenta. PS-particles are synthetic polymers and abundantly detectable in the environment and in human food chain [33, 34]. The significance to study these particles in pregnancy is given by reported effects on the reproductive system in rodents in vivo [35]. Further, they can be easily synthesized in a wide range of sizes with distinct surface functionalization, they are perfectly suited as model particles to study effects of the particle surface characteristics on biological parameters [36–38]. Ex vivo perfusion studies of human placental tissue were performed by using four culture media differing in protein and supplement composition. To reveal differences in the protein corona composition on PS-particles before and after tissue exposure in the fetal and maternal circulation we subjected the PS-particle corona to proteome analysis. The transfer of 80 nm PS-particles had been studied, which prompted us to use these particles in our study as well [11, 20].

## Results

### Characterization PS-particles

To analyze particle sizes in heterogeneous formulated media, nanoparticle tracking analysis (NTA) was carried out [39]. Table 1 summarizes the determined sizes in the different media compositions. The smallest mass-median-diameter (D50) particle sizes (96.4 ± 0.4 nm) were recorded in phosphate buffered saline (PBS), which served as a reference. Particle sizes increased in all tested media compared to PBS. The highest PS-particle size 148.8 (± 24.1) nm was measured in plasma medium. The other three supplemented media showed an increase in D50 particle sizes between 25–44 nm (Table 1). NTA detected several sub-fractions of PS-particles. Table 1 highlights specifically the size of PS-particles with the highest abundancy in the respective medium. The total particle size distribution of individual medium is presented in the supporting information (Additional file 1: Figure S1). Zeta-potential of PS-particles was recorded at -27.63 (± 0.72) mV in PBS. In the four perfusion media the zeta potential increased differently but remained negative in every setting.

**Table 1:** Composition of media and characteristics of PS-particles in respective fluids

Media	Control	Plasma*	HSA	IgG	PBS ref
Dextran (w/v%)	1	0	0	0	0
BSA (w/v%)	0.5	0	0	0	0
HSA (w/v%)	0	4	4	0	0
IgG (w/v%)	0	0	0	1	0
Plasma (v/v%)	0	8.6	0	0	0
D50 (nm)	135.5 (± 3.7)	148.8 (± 24.1)	116.3 (± 6.2)	129.8 (± 2.3)	96.4 (± 0.4)
Size main particle fraction (nm)	115	107	104	105	89
Zeta potential ± SD (mV)	− 13.23 (± 0.91)	− 9.61 (± 0.85)	− 7.05 (± 0.64)	− 3.54 (± 0.63)	− 27.63 (± 0.72)

^*^ + 33 µg/ml of the thrombin inhibitor Argatroban

### Plasma alters maternal to fetal transfer of PS-particles

**Fig. 1:**
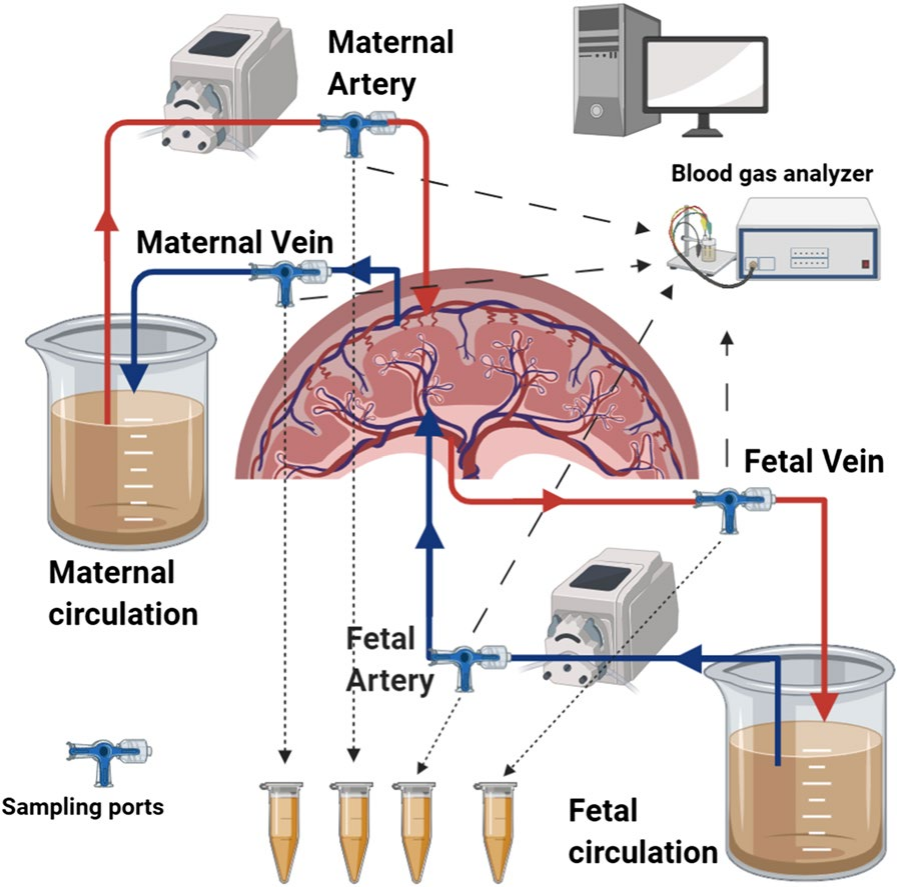
Scheme of the ex vivo placental perfusion setting. (with the use of BioRender.com)

Ex vivo placental transfer experiments with de novo protein coated PS-particles were performed by using the placental perfusion model. In the applied setting PS-particle concentration in maternal and fetal in (artery) and outflow (vein) to and from the placental tissue was recorded (Fig. 1).

**Fig. 2:**
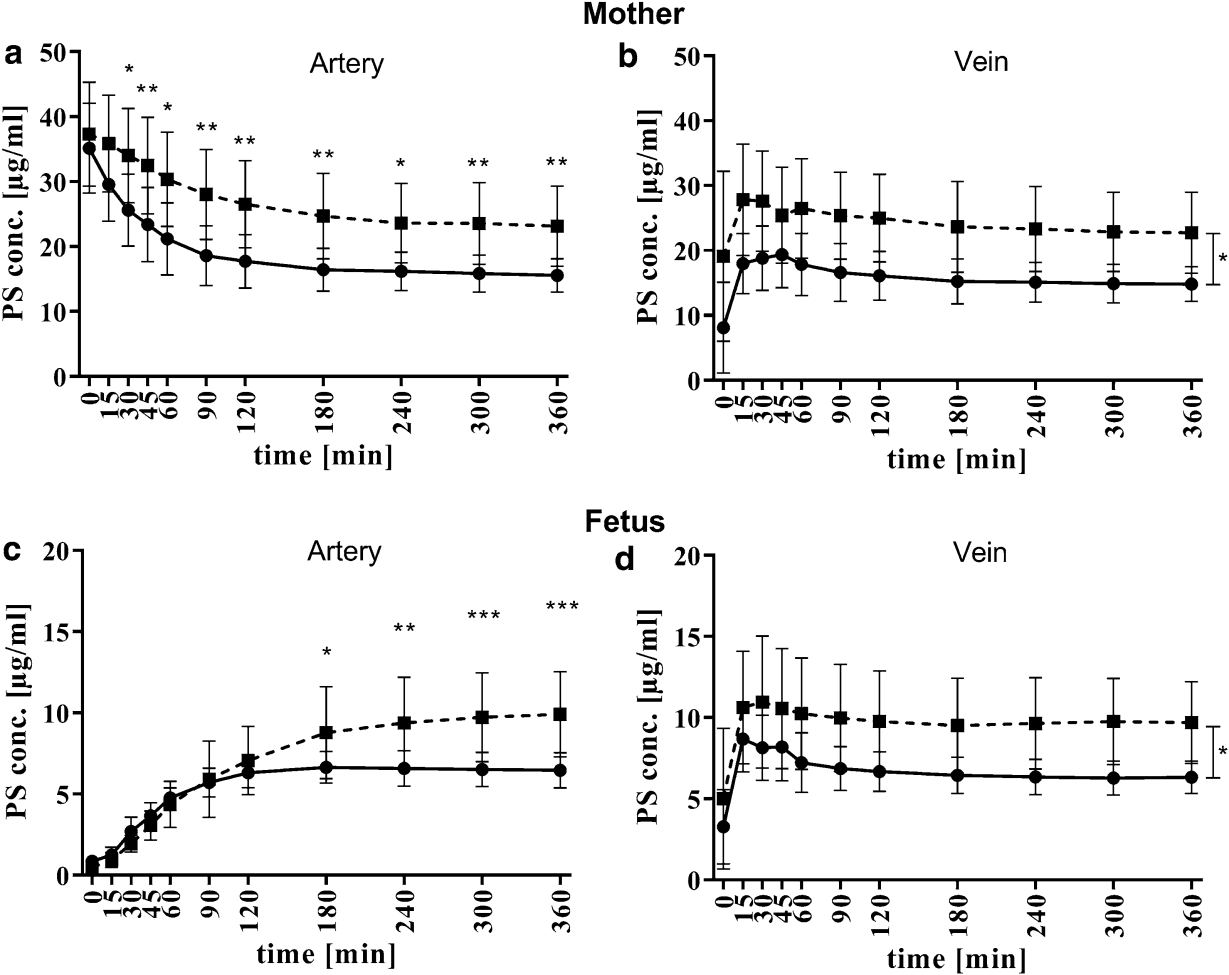
Time kinetics of PS-particles concentrations in maternal and fetal compartment using control- (continuous line, n = 5) or plasma medium (dashed line, n = 5). Each graph shows data obtained from different sampling ports: **a** maternal artery, **b** maternal vein, **c** fetal artery, and **d** fetal vein. Statistical analysis by using a linear mixed model was performed. Significant interaction effects (time, media) are displayed above distinct time points. Significant main effect (media) is indicated to the right of the graphs (**b**, **d**). Statistically significant differences are depicted as: *p < 0.05, **p < 0.01 and ***p < 0.001. Data are presented as mean ± SD

In first set of experiments a concentration of 40 µg/ml fluorescent labeled PS-particles was used in control and plasma media (Fig. 2). The difference in PS-particle concentration was the main determinant by comparing control and plasma media in both circulations. Already after 30 min we detected a significant difference in PS-particle concentrations in the maternal artery. After 6 h of perfusion the levels remained significant different by 23.2 (± 5.5) µg/ml in the plasma and 15.5 (± 2.3) µg/ml PS-particles in the control medium (p = 0.0074, Fig. 2a). Interestingly, during the first 2 h of organ perfusion the difference of absolute PS-particle levels increased constantly between the used media while this difference remained nearly constant between 2- to 6 h.

The detected PS-particle concentration in the maternal vein—representing the back flow from the placenta to the mother, was lower than in the artery within the first 60 min however, converged over time. These findings indicate a saturation of PS-particle concentration in/at the placenta after 2 h of perfusion independently of used medium. The final PS-particle concentrations in the maternal vein reached 22.7 (± 5.6) µg/ml and 14.8 (± 2.4) µg/ml for plasma and control medium, respectively [(p = 0.0056), Fig. 2b].

Strikingly, a PS-particle concentration difference between the two media appeared in the fetal vein, which represents the flow from the placenta to the fetus, already after 15 min (Fig. 2d). Independently of the medium PS-particle levels reached a maximum in the fetal vein after 15–30 min of tissue perfusion. Thereafter, PS-particle levels declined over time and reached a final concentration of 9.8 (± 2.8) µg/ml and 6.7 (± 1.1) µg/ml in plasma and control medium, respectively (p = 0.014).

In contrast, the back flow from the fetus to the placenta (fetal artery) of PS-particles progressed similarly and slowly within the first 90 min media independently. This observation is explainable by a PS-particle dilution in the respective medium. Thereafter, the PS-particle transfer kinetics changed significantly and the differences were related to the used cell culture medium and the proportion of differences similar to the fetal vein. In plasma medium a final concentration of 9.9 (± 2.3) µg/ml was observed levels and a final concentration of 6.5 (± 1.0) µg/ml. in control medium (p = 0.0003, Fig. 2c).

We located potential PS-particles in perfused placental tissue by transmission electron microscopy. Organelles containing PS-particles were located mainly in the syncytiotrophoblast layer which represents the first maternal–fetal barrier of the placenta (Additional file 1: Figure S2). No particles were detectable in the thin sections of the remaining tissue areas like the placental stroma or around the feto-placental-endothelium.

### Increased abundancy of albumin and immunoglobulin on PS-particles in the fetal compartment

The composition of protein corona on isolated PS-particles from plasma medium before and after tissue perfusion was analyzed by shotgun proteomics. Based on LC–MS/MS analysis, the qualitative and quantitative composition of the PS-particle corona before and after passage across the placenta was assessed (List of protein identified in each compartment is presented in Additional file 2: Tables S5, S6). In general, these results demonstrated a qualitative change of protein composition of the PS-particle corona in plasma medium in the maternal and fetal circulation. We found 129 different proteins on PS-particles in plasma medium which were isolated before perfusion. The relative abundancy of proteins changed significantly on particles which crossed the placenta (93 different proteins). Particles isolated from maternal circulation after perfusion contained 642 different proteins in their corona (Additional file 1: Figure S3, Additional file 2: Tables S5, S6).

**Fig. 3:**
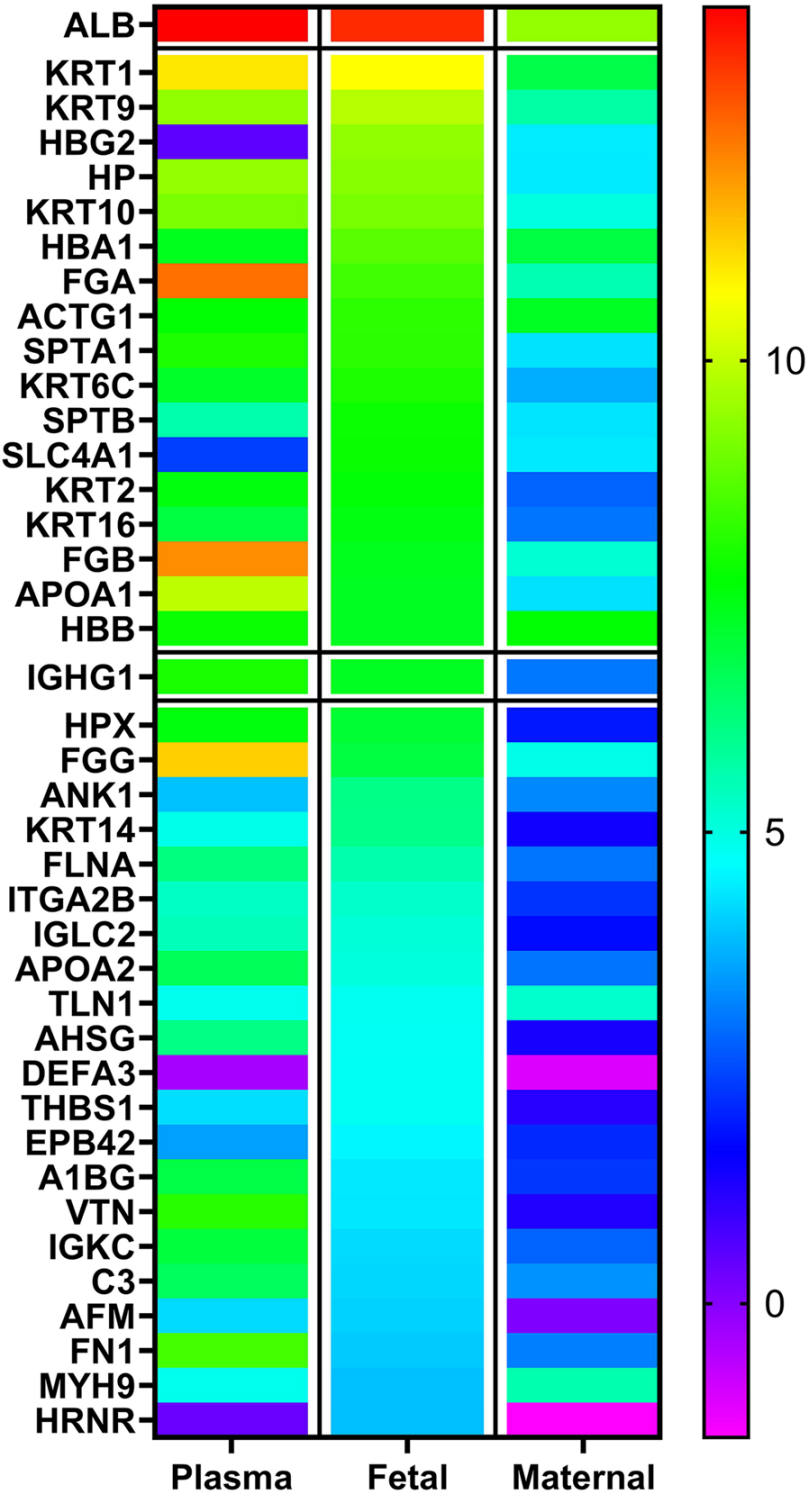
Heatmap displaying normalized abundancy of proteins on PS particles. The color scale illustrates the relative level of each protein on particles in plasma and maternal media compared to the 40 most abundant ranked proteins on particles in the fetal circuit. The different colors indicate the level of protein abundancy. The highlighted proteins were selected for detailed analysis (albumin and IgG, framed boxes)

Subsequently, we analyzed the data quantitatively after post hoc normalization. Therefore, we normalized the protein intensity to median protein intensity signals in each respective LC–MS/MS run in order to detect enriched proteins on PS-particle coronas in the fetal circulation (Additional file 1: Figure S4. Additional file 3: Table S7). Both plasma proteins-albumin (HSA) and IgG with a relative intensity of 39% (± 10) and 0.6% (± 0.2) respectively, were significantly increased by 29% and 0.5% on transferred PS-particles compared to maternal remaining PS-particles (p = 0.00719, q = 0.00473, and p = 0.00123, q = 0.00291) (Fig. 3. Additional file 1: Figure S5).

### Plasma albumin drives the PS-particle transfer across the placenta

**Fig. 4:**
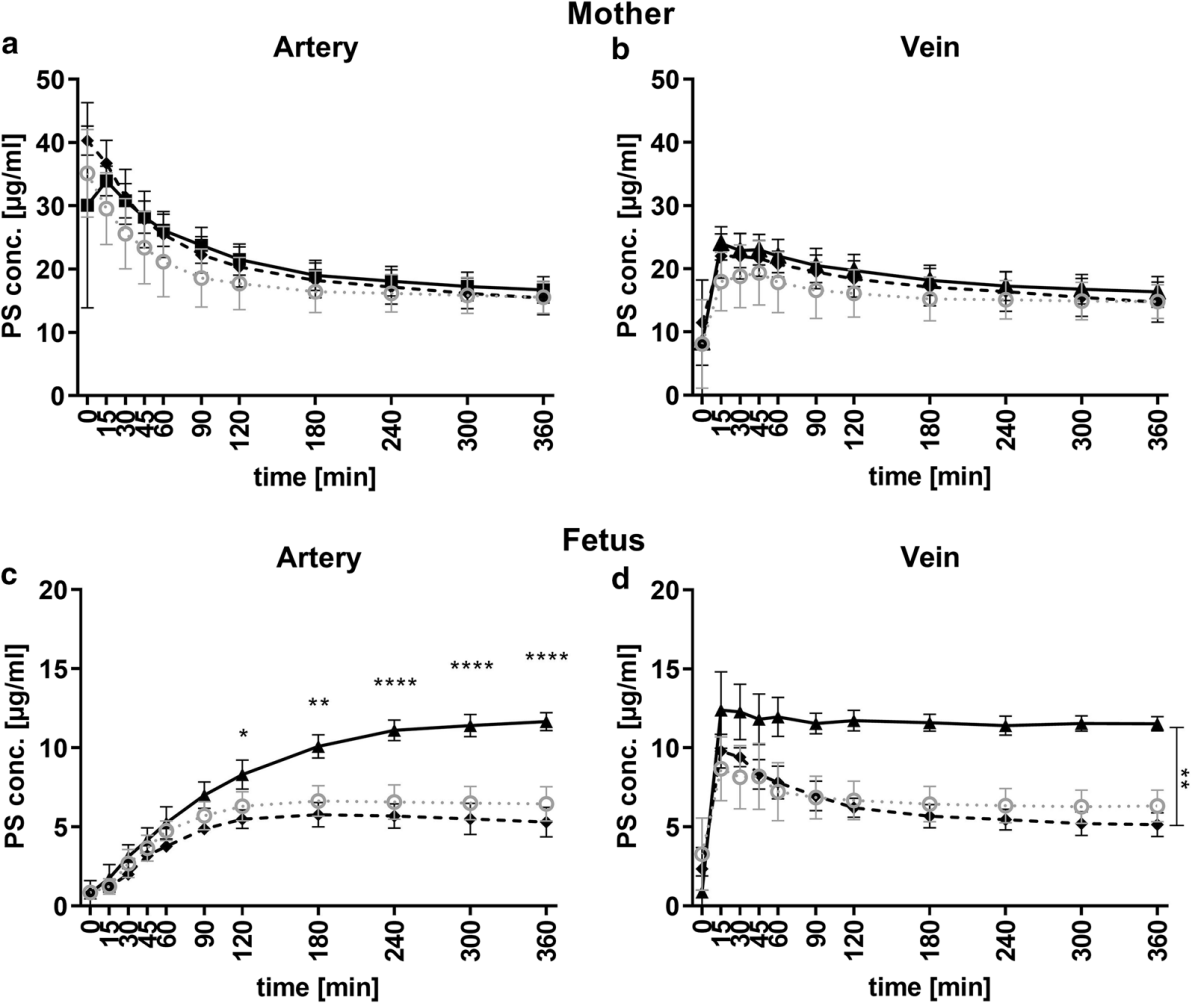
Time kinetics of PS-particle concentrations in maternal and fetal compartment using HSA- (continuous line, n = 5), IgG medium (dashed line, n = 3) or control medium (dotted grey line, n = 5). Each graph shows data obtained from different sampling ports: **a** maternal artery, **b** maternal vein, **c** fetal artery, and **d** fetal vein. Statistical analysis was performed by a linear mixed model. Significant interaction effects (time, media) are displayed above distinct time points (**c**). Significant main effect (media) is indicated to the right of the graphs (**d**). (Statistically significant differences are depicted as: *p < 0.05, **p < 0.01 and ****p < 0.0001. Data are presented as mean ± SD

Based on the protein analysis of the transferred PS-particles medium with HSA and IgG were formulated and applied in the perfusion model. In maternal circulation HSA and IgG showed a similar kinetics of PS-particle levels, irrespective of the sampling port (Fig. 4a, b). In comparison to control medium, a time-delayed decrease of PS-particle levels was observed with a similar final concentration of 16.7 (± 1.9) µg/ml for HSA and 15.5 (± 2.2) µg/ml for IgG medium (Fig. 4a). Final concentrations in the maternal vein of 16.3 (± 2.2) µg/ml for HSA and 14.7 (± 2.6) µg/ml for IgG medium were measured (Fig. 4b) after 6 h perfusion.

Media supplemented with 40 g/l HSA showed a higher materno to fetal transfer rate across the placenta. In the fetal vein a difference of PS-particles were detectable after 15 min between HSA and IgG medium (p = 0.0015, Fig. 4d) with a final concentrations of 11.7 (± 0.6) µg/ml in HSA and 5.1 (± 0.6) µg/ml in IgG medium. Both proteins passaged the tissue fast and achieved a steady-state after 15 min. The detailed statistical analyses are presented in the supporting information (Additional file 1: Tables S1–S4).

We detected a final concentration of 11.6 (± 0.5) µg/ml in the fetal artery using HSA medium in contrast only 5.3 (± 0.8) µg/ml IgG was detected in the respective medium(Fig. 4c). The PS-particle concentration in fetal artery during HSA experiments was significant higher than PS-particle concentrations in the other media after 4 h (control p < 0.0001, plasma p < 0.0461, IgG p < 0.0001).

### Protein composition of the medium determines recovery of PS-particles

**Fig. 5:**
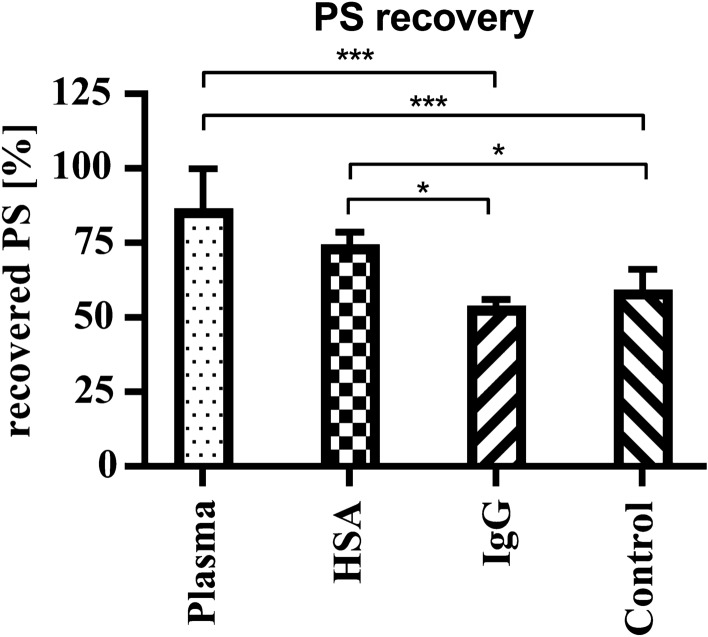
Recovery of PS-particles as a function of applied media. Total PS-particle levels of maternal and fetal reservoirs are normalized to applied PS-particle concentration in the maternal reservoir (40 µg/ml). Data was analyzed with One-Way-ANOVA and Holm-Sidak´s multiple comparison test in Graph Pad Prism. Data are presented as mean ± SD

The recovery rate of PS-particles differed between HSA- (74.5 ± 4.1%) and plasma medium (86.7 ± 13.3%) compared to control- (59.4 ± 6.7%) and IgG medium (54.1 ± 2.1%) indicating that the load of proteins on distinct PS-particles may account for this differences (Fig. 5). The most significant differences of PS-particle recovery were found between plasma medium to control- and IgG medium. (p = 0.0007, p = 0.0006 respectively).

## Discussion

Here we explored if and how plasma proteins may alter the transfer of unmodified PS-particles across the placental barrier. Our study showed for the first time that the protein corona composition of 80 nm PS-particles significantly changes upon crossing the human placenta. To better understand the effect of corona formation and composition on NP transfer across intact biological barriers, selected methodologies are likely to become important in this research area. The research presented here was carried out by using the ex vivo placental perfusion model, which can be considered as golden standard for transfer studies [40]. The benefits are the physiological integrity of the tissue, and the utilization of a dynamic flow setting compared to cell culture approaches. In biological medium, NP interact with proteins, lipids, and even biological metabolites. This fact is of particular importance as the adsorption of proteins on the surface of NP leads to a number of consequences such as an altered biological reactivity of the particles [6]. Therefore, besides an intact barrier, a well-defined and characterized cell culture medium is a prerequisite to investigate NP transfer. The medium which served herein as control is similarly composed as it has been used frequently [11, 41]. For PS-particle corona formation, we chose plasma and not serum, as physiological fluid since it contains proteins of the coagulation cascade, thereby resembling more closely in vivo conditions [12, 42, 43].

We decided to use only one size of PS-particles without any functionalization because 80 nm particles easily passage the placenta in opposite to many other applied NP, as previously described elsewhere [11, 44]. Moreover, coronas around two different sized neutral PS-particles are very similar indicating that the molecular (i.e. hydrophobicity) properties and applied biological fluids may account more significantly than the size of plain PS-particles to the composition of the corona [45]. The size and zeta potential of the particles were determined in the respective medium (see Table 1). As we expected to apply particles with heterogeneous sizes, nanoparticle tracking analysis measurements were performed as suggested for such complex matrices [39]. A clear biological corona formation expressed as an increase in PS-particle size compared to the PBS reference was observed [36]. However, our obtained data does not allow us to discuss the formation of mono- or multilayers of proteins around the particles [46]. The particles with the highest concentration called main peak (e.g., for PBS ~ 89 nm, Additional file 1: Figure S1), represent the majority of particles which are in contact with the placental tissue. In all protein-containing media, the main size peaks are in a range of 104–115 nm, clearly suggesting protein adsorption to the particle. In addition, PS-particle sizes below the PBS particle size were detected, implying the presence of protein or stabilizer aggregates in plasma and HSA medium. Peaks bigger in size than the main peak suggests formation of PS-particle aggregates (Additional file 1: Figure S1). As zeta potential may be influenced by other factors like ionic strength in the media and not solely by adhering molecules on the PS-particle surface [47] observed changes (see Table 1) cannot be linked as proof of corona formation in the different media. Together, these results illustrating the extreme care one needs to take with sample characterization before such studies.

Kinetics of protein adsorption on the PS-particle surface can be influenced by several factors and are continuously changing in respect of medium and time [1, 43]. When plasma proteins were applied between 3 and 80% of plasma, Monopoli et el., observed that bound proteins to NPs varied with plasma concentration while relative amounts of some proteins increased with higher plasma levels [48]. Although all corona formation of the particles was done in full plasma, we are aware of the limitation of the chosen experimental setting by using a rather low plasma concentration during perfusion of the tissue. This is a consequence of the used ex vivo model, which cannot be operated with higher plasma levels because of viscosity and coagulation problems. However, our approach aimed to identify protein candidates, probably facilitating placental transfer of PS-particles in plasma medium. Based on herein used sample preparation for proteomic analysis, we likely detected proteins that are strong adhering to the particles. We could confirm that albumin, immunoglobulins and apolipoproteins are major proteins of the corona [49–51]. Loosely adhering proteins to the particles were likely not detected given by the chosen isolation method [1, 52, 53].

Many studies on protein corona were focused on NPs that are incubated for a specific time in biological fluids or complex media [36, 54]. In addition to this classical approach, our approach allowed us to isolate and to characterize corona coated PS-particles after they interacted with the placenta or even passaged the tissue. Unexpectedly, we detected a significant increase in the number of proteins on isolated PS-particles which were in contact with the placenta compared to plain particles in plasma medium (642 vs. 129 proteins, Additional file 1: Figure S3). This observation may suggest recurrent PS-particle uptake and re-exocytosis at the placental barrier and thereby an enrichment of corona proteins during this process. The increase variety of intracellular proteins associated with the corona further supports this idea. Another reasonable explanation for this protein variety could be the secretion of placental derived proteins, which may accumulate on the particle in the recirculating medium over time. In total this finding argues at least partly for a non-specific cellular interaction of the PS-particles depending on the amount of proteins rather than the presence of distinct proteins on the PS-particle which was already shown by Ehrenberg et al. on endothelial cells in vitro [55].

Given the small size of PS-particles, it is very likely that they translocate across membrane barriers of an organ. Nanoparticles with a diameter of around 100 nm can enter cells [56]. Looking at the number of corona proteins on transferred PS-particles in the fetal circulation, we see a striking reduction of individual proteins compared to the number of corona proteins in the maternal circulation (642 vs. 93 proteins, Additional file 1: Figure S3). This decrease in corona protein diversity after tissue transfer may result from specific intracellular processes, not yet been elucidated. For example, intracellular pH-changes may alter the affinity of specific proteins to the particle resulting in changes in protein binding after placental transfer [57].

**Fig. 6:**
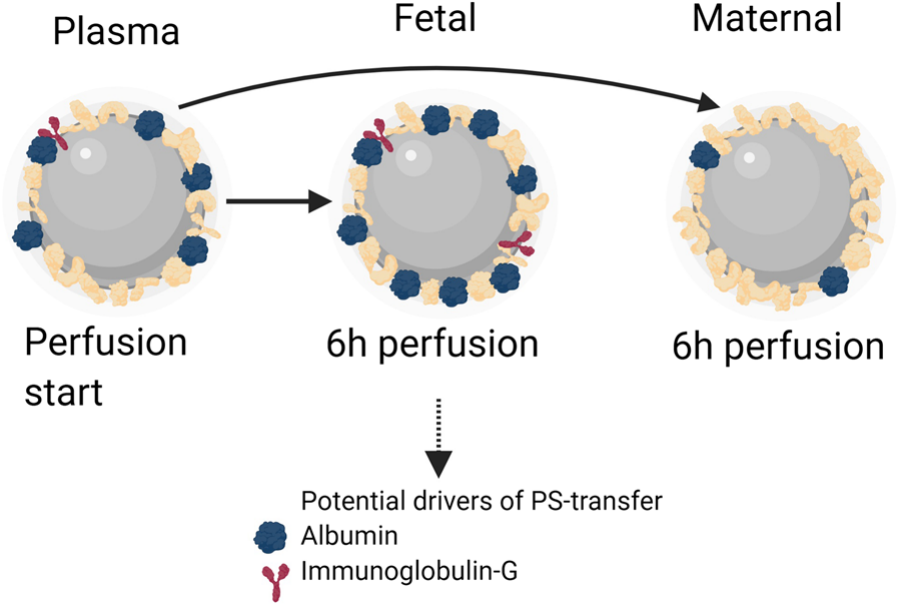
Scheme illustrating changes of protein corona during placental perfusion experiments. The PS-particle protein corona changed significantly in the number and abundancy of proteins depending on the perspective. PS-particles isolated from the fetal circulation showed a decreased protein diversity after 6 h, but relative higher protein abundancy for some specific proteins e.g. albumin and IgG. Particles isolated from the maternal circulation showed a major increase in the total number of different corona proteins (with the use of BioRender)

The quantitative comparison of the corona compositions before and after placental passage led us to conclude that HSA and IgG since both were significantly enriched on PS-particles, are potential drivers of the increased materno-to-fetal PS-transfer as proposed in Fig. 6.

Both proteins were found on protein coronas of PS-particles before [1]. Albumin was tested to functionalize nanomaterials to enhance cellular uptake, target cells, or study transfer in several models [58–60]. Additionally, caveolae-mediated endocytosis of 100 nm fluorescent PS-particles was shown to be dependent on the abundance of albumin on the corona surface, supporting the idea that it might be an important driver for cellular uptake of PS-particles [61]. Our additional results with HSA containing media are an extension to studies demonstrating NP transfer by using cell barrier systems in the presence of albumin [58, 59].

It was shown that plain HSA might cross the placenta in negligible quantities [62]. Therefore, our observed HSA dependent PS-particle transfer may be different from physiological HSA processing at the placental barrier. Nevertheless, corona associated albumin can change its conformation, which may directly influence particle transfer in our experiments [57, 63]. Whether these results on 80 nm PS-particles are transferable to smaller or larger PS-particles needs to be investigated in future.

Furthermore, since IgG is known to be transferred from the mother to the fetus during pregnancy, we expected it to be a promising candidate for mediating particle transfer [64]. In addition, the accumulation of multiple keratins in the fetal PS-particle corona may indicate tissue passage rather than an accidental bypass of the barrier. Apolipoproteins are a class of proteins that are often found in the corona of NPs and discussed as an additional plasma protein, which may assist in transport of nanomaterials across barriers [65]. It is worthy of note that apolipoprotein A1 [beside apolipoprotein E and apolipoprotein (a)], which represents the main structural protein of HDL, is also significantly enriched in the corona of transferred PS-particles in the fetal circulation (Additional file 1: Figure S5).

Different media compositions were used in placental perfusion studies of nanomaterials, depending on the addressed research question [30, 41, 66, 67]. Our findings demonstrate, for the first time, a protein-specific and medium dependent PS-uptake into and/or adherence of particles to placental tissue. Interestingly, a similar final PS-particle concentration was observed in the maternal circulation with control, HSA, and IgG medium. The similar PS-particle uptake in HSA and IgG medium can be explained by a receptor facilitated binding of HSA and/or IgG at the placental barrier, as discussed below [68–70].

By comparing our observation of placental PS-particle transfer in control medium to already reported results, we noticed a lower transfer rate [11]. This inconsistency may be due to a lower albumin concentration, which we used in our sets of experiments as we demonstrated that albumin containing media is linked to an enhanced PS-particle transfer rate. One study suggested an inverse relation of PS-particle agglomeration and albumin concentration in the surrounding medium, which could lead to the observed decreased transfer [57]. Additionally, the flow rates during perfusion of the tissue deviate from each other. In the work of Wick et al., a maternal flow rate of 12 ml/min and a fetal flow rate of 6 ml/min was applied compared to our flow rates of 9 ml/min and 3 ml/min, respectively. These differences may impact shear stress and filtration pressure within the tissue, which ultimately could impact PS-particle cell interaction [71]. Notably, a baseline transfer of PS-particles across the placenta was observed in all media and independently of the addition of proteins, indicating a second underlying mechanism in this transfer process. Even as we could detect PS-particle transfer across the placenta our data does not answer questions on potential harm of these PS-particles in the placental tissue.

## Conclusion

The transfer of NPs across biological barriers is specifically dependent on both the properties of the particles and used experimental model. Based on our findings, we conclude that a dynamically forming protein corona influences significantly the transfer of 80 nm PS-particles across the human placental barrier. By using an ex vivo barrier model we see that the initial composition of PS-particles in the maternal circulation is not predictive for their transfer characteristics and their functionality once beyond the placental barrier. It therefore appears plausible to state that binding of certain proteins on PS-particles contribute to the onset of deregulation of cellular pathways within the tissue. The underlying mechanisms and if human placenta specific are unknown but call for further studies. In summary, the composition of the corona on NPs which, is dependent on the applied biological environment, dictates the overall biological reactivity. To understand the dynamics of this complex NP-tissue interaction provide novel insights into key properties of these particles that can be explored for future applications of such nanomaterials.

## Methods

### Perfusion media

In this study, four different formulated media were used, all supplemented with 40 µg/ml 80 nm fluorescent-labeled plain PS-particles (Kisker, Steinfurt, Germany) (Table 1). All media were based on Dulbecco’s modified eagle medium (DMEM) (Thermo Fischer Scientific, Vienna, Austria) and buffered by earls buffered salt solution (EBSS) (Merck, Darmstadt, Germany) in a ratio of 2 to 1 respectively, supplemented with 1.33 g/l d-Glucose and 250 mg/l Amoxicillin (Merck, Darmstadt, Germany). The control medium was supplemented with 40FP dextran (SERVA Electrophoresis GmbH, Heidelberg, Germany) and 0.5% BSA (Merck Darmstadt, Germany) as frequently used [11]. PS-particle stock solution, [1.2 ml of 1% (w/v)] was incubated with (i) 26 ml human female plasma, (ii) 60 ml of HSA (Octapharma, Vienna, Austria) solution (200 g/l), (iii) 30 ml of polyclonal human IgG (CLS Behring, Vienna, Austria) solution (100 g/l) or (iv) 300 ml of the control medium, for 15 min at 37 °C to allow corona formation. We made use of the experience that in biological fluids corona formation of NPs changes significantly within the first 15 min [1, 8]. The final concentration of respective perfusion medium was: (i) 8.6% plasma, (ii) 40 g/l HSA and (iii) 10 g/l IgG < media concentration or in 300 ml as final maternal medium. To mimic a physiological corona formation instead of serum, plasma, which contains proteins of the coagulation cascade, was administered [12, 42, 60]. Argatroban (0.033%, Mitsubishi Pharma, Vienna, Austria), a direct thrombin inhibitor, was added to inhibit the coagulation cascade, since it may influence the corona composition [12]. The medium in fetal circulation was identical to the maternal medium but without PS-particles.

### Dual ex vivo perfusion of a placental cotyledon

The herein used model meets several conditions similar to in vivo, e.g., applied flow at the tissue barrier, including the formation of shear stress and filtration pressure. Some of that determinants differ significantly from traditionally used static cell culture systems and may contribute to biocorona formation and finally further affect the interplay between PS and tissue [10]. To achieve the closest proximity to an in vivo situation the model setting was introduced as outlined in Fig. 1. In order to maintain colloid osmotic pressure and total protein levels closer to physiology, higher normal concentrations of HSA were used in this setting [72]. The employed plasma concentration was selected according to the suggested ratio of particle surface and plasma levels and experimental practicability [73]. Besides, this ex vivo approach allowed us to analyze the proteome of respective biocoronas on the PS particles before and after tissue exposure in the maternal and fetal circulation distinctly.

All experimental setups were executed based on literature [74, 75]. Briefly, the perfusion experiments were structured in three phases. First, maternal and fetal circulations were set up by cannulating the intervillous space and respective chorionic vessels and were connected to the corresponding recirculating pump systems. Maternal and fetal flow rates were adjusted to 9 ml/min and 3 ml/min, respectively. In order to remove the blood, tissue was washed with the respective medium for 30–60 min. Second, the medium was changed to PS containing maternal and corresponding fetal medium with a recirculating setup. Sample ports at the maternal and fetal side were applied to collect 1.5 ml of samples onward starting tissue perfusion (0 min) and after 15, 30, 45, 60, 90, 120, 180, 240, 300, 360 min. Third, a quality control phase was executed by exchanging the PS medium to medium containing 100 µg/ml antipyrine without PS in the maternal circulation. Samples were drawn at 0, 10, 20, 30 min from maternal artery and vein as well as fetal vein to quantify antipyrine. Since this compound exerts passive membrane diffusion properties, the fetal to maternal transfer ratio of antipyrine predicts the surface exchange area between both circulations. To monitor the quality and viability of the placental tissue during the experiments O_2_ and CO_2_ tension, glucose and lactate concentration, as well as pH, was measured at discrete time points from all sampling ports by using an ABL 800 basic (Radiometer, Copenhagen, Denmark) blood gas analyzer. If necessary, pH was adjusted with citric acid to match physiological ranges. Additionally, fetal vessel backpressure was monitored using a digital catheter pressure sensor (Millar, Houston, TX). Perfusion experiments were performed in the dark to avoid PS bleaching. All experiments were validated with minor modifications to the literature [76]. A successful perfusion experiment met several criteria:

(i) circulation in the fetal-placental compartment was established within 30 min after placental delivery; (ii) fetal volume recovery of 95% within 10 min during the blood washout; (iii) oxygen transfer from maternal to fetal side; (iv) volume deviation < 24 ml between maternal and fetal reservoir at the end of PS transfer experiments; (v) antipyrine fetal to maternal ratio of at least 0.3 within 30 min perfusion time; (vi) glucose consumption; (vii) lactate formation; (viii) pH between 7.2 and 7.5; (ix) fetal vessel backpressure < 65 mbar.

### Quantification of antipyrine

Antipyrine (Sigma Aldrich, Steinheim, Germany) was quantified by high-performance liquid chromatography (HPLC) according to Annola et al. [77]. Samples were analyzed by a liquid chromatography system (Knauer, Germany, autosampler 3950, degasser 5050, pump 1050, and UV-detector 2500 wavelength of 255 nm) and separated before by an Aquasil C18 (150 × 2.1, 5 µm) column (Thermo Scientific, Vienna, Austria). Acetonitrile and 20 mM K_2_HPO_4_ (Merck, Darmstadt, Germany 1:1 mixture) was applied as mobile phase by an isocratic flow of 0.2 ml/min at room temperature.

### Zeta potential and particle size determination for the particles

Particle sizes were determined with a NanoSight NS 300 instrument (Malvern Instruments Ltd., Worcestershire, UK). The system was operated with a 488 nm laser at slider gain of 15, camera levels of 9 for the media except for plasma medium where camera level was 5. The PS particles were sonicated for 3 min, subsequently incubated for 15 min at a concentration of 400 g/ml with the respective medium to allow protein corona formation.

For zeta potential determination 80 nm PS-particles were sonicated for 3 min, diluted in the perfusion media or PBS to a concentration of 1 µg/ml, and incubated for 15 min at room temperature. Zeta potential was determined with a Zetasizer Nano ZS (Malvern Instruments Ltd., Worcestershire, UK) at 25 °C in disposable folded capillary cell (Malvern Instruments Ltd., Worcestershire, UK) after 2 min of equilibration time. Measurements were performed three individual times with a minimum of 10 replicates and a maximum of 100 runs in each measurement.

### Quantification of fluorescence

For quantification of fluorescent PS particles in the perfusion samples, the collected perfusates were vortexed, and 200 µl were transferred to non-binding black F-bottom 96 well microplates (Greiner bio-one, Kremsmünster, Austria). Each well was measured at an excitation of 544 nm and an emission of 590 nm by using a FluoStar Optima fluorescence plate reader (BMG Labtech, Ortenberg, Germany). The readout was compared to freshly prepared standards (1, 5, 15, 35, 75 µg/ml) in the corresponding medium for quantification.

### Sample preparation of collected maternal and fetal perfusates

To investigate the differences of corona proteins on the PS surface, 6 ml of maternal and fetal medium after 360 min tissue perfusion and 2 ml of non-perfused PS-medium pre-incubated with plasma were collected and processed. To remove cellular debris and un- or weakly bound proteins, all samples were centrifuged for 10 min at 5000*g* at 4 °C. The supernatant was collected and immediately loaded on a sucrose gradient, re-centrifuged followed by three washing steps as described elsewhere [73]. All pellets were stored at − 80 °C until LC–MS/MS analysis.

### LC–MS/MS analysis

PS-particles bound corona protein samples were re-dissolved in TFE-digestion buffer (25% 2.2.2-trifluoroethanol (TFE) in 50 mM Tris–HCl, pH = 8.5) and subsequently centrifuged (15,300*g*, 4 °C, 10 min.). Reduction and alkylation were carried out using 10 mM tris(2-carboxyethyl) phosphine (TCEP) and 40 mM chloroacetamide (CAA) for 1 h at 37 °C. Samples were diluted to 10% TFE with 50 mM ammonium bicarbonate and digested using rLysC in a protein: enzyme ratio of 100:1 at 37 °C for 3.5 h, followed by modified trypsin protein: enzyme ratio of 50:1 at 37 °C overnight (both enzymes from Promega, Germany). The enzymatic reaction was stopped by adding 5% formic acid to reach 0.1% final concentration. The resulting peptide solution was then filtered through a 10 kDa cut-off filter to remove PS and similar peptide amounts were injected to align base peak chromatograms into a nano-HPLC (Dionex Ultimate 3000) equipped with a C18, 5 µm, 100 Å, 5 × 0.3 mm enrichment column and an Acclaim PepMap RSLC nanocolumn (C18, 2 µm, 100 Å, 500 × 0.075 mm) (all Thermo Fisher Scientific, Austria). Samples were concentrated on the enrichment column for 6 min at a flow rate of 5 µl/min with 0.1% heptafluorobutyric acid as isocratic solvent. Separation was carried out on the nanocolumn at a flow rate of 300 nl/min at 60 °C using the following gradient, where solvent A is 0.1% formic acid in water and solvent B is acetonitrile containing 0.1% formic acid: 0–6 min: 4% B; 6–94 min: 4–25% B; 94–99 min: 25–95% B. 99–109 min: 95% B; 109.1–124 min: 4% B; The maXis II ETD mass spectrometer (Bruker, Austria) was operated with the captive source in positive mode employing the following settings: mass range: 200–2000 m/z, 2 Hz, capillary 1300 V, dry gas flow 3 L/min with 150 °C nanoBooster 0.2 bar, precursor acquisition control top17 (CID).

### Mass spectroscopy data processing

The LC–MS/MS data were analyzed by Data Analysis software (Bruker Austria GmbH, Vienna, Austria), using the Sum Peak algorithm, and by MaxQuant 1.5.8.3 searching the public Swissprot database with taxonomy homo sapiens (downloaded on 02.03.2017) and common contaminants (20233 sequences). Carbamidomethylation on Cys was entered as a fixed modification, oxidation on methionine as variable modification. Detailed search criteria were used as follows: trypsin, max. missed cleavage sites: 2; search mode: MS/MS ion search with decoy database search included; precursor mass tolerance ± 0.006 Da; product mass tolerance ± 40 ppm; acceptance parameters for identification: 1% PSM false discovery rate (FDR); 1% protein FDR. Besides, an intensity based quantitation including the match between runs feature of MaxQuant was performed [78] requiring a minimum of 2 quantified razor and unique peptides. The software Perseus version 1.6.12.0 was used for further data processing. Intensities were log2 transformed and reduced to proteins detected in all samples of at least one sampling cohort (4 valid in a group of 4, plasma, maternal or fetal).

We chose a conservative interpretation strategy meaning that we only included proteins which were present on all isolations of the respective sampling group. Analysis of qualitative protein distribution between groups was performed with Venny (Oliveros, J.C. (2007–2015) Venny, https://bioinfogp.cnb.csic.es/tools/venny/index.html). Intensity data was median normalized for each respective LC–MS/MS run. For missing protein values in the corresponding cohorts, intensity values were imputed. Briefly, missing values were replaced with random values taken from a shifted Gaussian distribution of all valid values (width of 0.3 and downshift of 1.8 separately for each column), in order to simulate an intensity value for low abundant protein groups. For statistical analysis, multiple t-testing corrected with permutation-based FDR method was used to identify significant proteins between groups [79]. To estimate the relative distribution of the significantly enriched proteins in the fetal coronas, relative intensity calculation was performed.

The mass spectrometry proteomics data have been deposited to the ProteomeXchange Consortium (https://proteomecentral.proteomexchange.org) via the PRIDE partner repository [80] with the dataset identifier PXD018160.

### Transmission electron microscopy

The placental tissues were perfused with 0.1 M sodium cacodylate buffer (Electron Microscopy Services, EMS, Hatfield, PA, USA), at pH 7.4. and subsequently fixed with 2% formaldehyde (Merck, Darmstadt, Germany) and 2% glutardialdehyde (Merck, Darmstadt, Germany). Tissue specimens of 1 mm^3^ were isolated and rinsed over night at 4 °C in perfusion buffer and post fixed in 2% osmium tetroxide in the same buffer. They were dehydrated in a series of graded ethanol (Merck, Darmstadt, Germany), placed into propylene oxide (Sigma-Aldrich, Steinheim, Germany) as an intermedium and embedded in TAAB embedding resin (TAAB, Aldermaston, UK). Leica UC 6 ultramicrotome (Leica, Vienna, Austria) was used to obtain 70 nm thick tissue sections. Tissue sections were visualized with a Zeiss EM 900 transmission electron microscope (Carl Zeiss MicroImaging GmbH, Oberkochen, Germany) at an acceleration voltage of 80 kV. Scale bars and structure indicating arrows were consecutively inserted to the images with ImageJ 1.50e [81].

### Statistical analysis

Descriptive statistics were used to analyze the data of particle quantification. Categorical results (perfusion time, maternal/fetal side, type of medium) are presented as absolute and relative frequencies, continuous data (PS concentration in µg/ml) as means, and standard deviations. A linear mixed model assessed the relationship between the quantity of transported PS and the independent variables (perfusion time, maternal/fetal side, type of media). The model included a random intercept for every successful placental perfusion experiment. Tissue perfusion time was considered as a repeated factor, and all the other variables were entered as a fixed effect. An auto-regressive heterogeneous covariance structure was used. The statistical analyses were performed with SAS software (version 9.4; SAS Institute. Inc., Cary, NC, USA). The graphs for data presentation and the recovery of PS particles after perfusion were analyzed with One-Way-ANOVA with a subsequent Holm-Sidak´s multiple comparison test in the software GraphPad Prism version 8.2. (GraphPad Software, San Diego, CA).

## Supplementary information

**Additional file 1: Figure S1.** Size distribution of 80 nm polystyrene particles. **Figure S2.** Electron microscopic images. **Figure S3.** Venn Diagramm. **Figure S4.** Volcano plot. **Figure S5.** Relative intensity of isolated fetal corona proteins. **Figure S6.** Albumin corona particle concentration in fetal circulation. **Table S1.** Statistics maternal arteries. **Table S2.** Statistics fetal arteries. **Table S3.** Statistics maternal veins. **Table S4.** Statistics fetal veins. References

**Additional file 2: Table S5.** Processed mean intensity of corona proteins LC–MS/MS data. **Table S6.** Raw LC–MS/MS data of detected corona protein intensity. Information Table S5. Information Table S6.

**Additional file 3: Table S7.** Log2 transformed median normalized intensity of LC-MS/MS detected corona proteins. Information Table S7.

## Data Availability

The datasets used and/or analysed during the current study are available from the corresponding author on reasonable request.

## Abbreviations

BSA: Bovine serum albumin
CAA: Chloroacetamide
D50: Mass-median-diameter
DMEM: Dulbecco’s Modified Eagle’s Medium
EBSS: Earle’s balanced salt solution
FBS: Fetal bovine serum
FDR: False discovery rate
HPLC: High-performance liquid chromatography
HSA: Human serum albumin
IgG: Immunoglobulin γ
LC–MS/MS: Liquid chromatography coupled to tandem mass spectroscopy
NP: Nanoparticle
NTA: Nanoparticle tracking analysis
PBS: Phosphate buffered saline
PS-particles: Polystyrene nanoparticle
TCEP: Tris (2-carboxyethyl) phosphine
TFE: 2.2.2-Trifluoroethanol
