# Supplementary material for: Plasma proteins facilitates placental transfer of polystyrene particles
Source: J Nanobiotechnology. 2020 Sep 9;18:128. doi: 10.1186/s12951-020-00676-5 (PMC7487953; doi:10.1186/s12951-020-00676-5)
Supplement: Supplementary file 1 — Additional file 1: Figure S1. Size distribution of 80 nm polystyrene particles. Figure S2. Electron microscopic images. Figure S3. Venn Diagramm. Figure S4. Volcano plot. Figure S5. Relative intensity of isolated fetal corona proteins. Figure S6. Albumin corona particle concentration in fetal circulation. Table S1. Statistics maternal arteries. Table S2. Statistics fetal arteries. Table S3. Statistics maternal veins. Table S4. Statistics fetal veins. References [file 12951_2020_676_MOESM1_ESM.docx]

Supporting Information

**Plasma proteins facilitates placental transfer of polystyrene particles**

Michael M. Gruber^+^, Birgit Hirschmugl^+,#^, Natascha Berger^+^, Magdalena Holter^†^, Snježana Radulović^∞ ,˟^, Gerd Leitinger˟, , Laura Liesinger^⁋,‡^, Andrea Berghold^†^, Eva Roblegg^⸸^, Ruth Birner-Gruenberger^‖,⁋,‡^, Vesna Bjelic-Radisic^+^, Christian Wadsack^+,#,*^

+ Department of Obstetrics and Gynecology, Medical University of Graz, Auenbruggerplatz 14, 8036 Graz, Austria

# BioTechMed-Graz, Mozartgasse 12/II, 8010 Graz, Austria

† Institute for Medical Informatics, Statistics and Documentation, Medical University of Graz, Auenbruggerplatz 2, 8036 Graz, Austria

∞Division of Molecular Biology and Biochemistry, Gottfried Schatz Research Center, Medical University of Graz, Neue Stiftingtalstraße 6/VI, 8010 Graz, Austria

˟ Research Unit Electron Microscopic Techniques, Division of Cell Biology, Histology and Embryology, Gottfried Schatz Research Center, Medical University of Graz, Neue Stiftingtalstraße 6/II, 8010 Graz, Austria

⁋ Diagnostic and Research Institute of Pathology, Diagnostic and Research Center for Molecular BioMedicine, Medical University of Graz, Stiftingtalstrasse 6, 8010 Graz, Austria.

‡ Omics Center Graz, BioTechMed-Graz, Stiftingtalstrasse 24, 8010 Graz, Austria

⸸ Institute of Pharmaceutical Sciences, Department of Pharmaceutical Technology and Biopharmacy, University of Graz, Universitätsplatz 1/EG, 8010 Graz, Austria

‖ Institute of Chemical Technologies and Analytics, Faculty of Technical Chemistry, Vienna University of Technology-TU Wien, Getreidemarkt 9/164, 1060 Vienna, Austria.

* Corresponding Author: Christian Wadsack PhD, Department of Obstetrics and Gynaecology, Medical University of Graz, Auenbruggerplatz 14, 8036 Graz, Austria. Email: christian.wadsack@medunigraz.at

**Table of Content**

Supporting Figure 1 - Size distribution of 80 nm polystyrene particles 4

Supporting Figure 2 - Electron microscopic images 5

Supporting Figure 3 – Venn Diagramm 6

Supporting Figure 4 – Volcano plot 7

Supporting Figure 5 - Relative intensity of isolated fetal corona proteins. 8

Supporting Table 1 - Statistics maternal arteries 9

Supporting Table 2 – Statistics fetal arteries 10

Supporting Table 3 – Statistics maternal veins 11

Supporting Table 4 – Statistics fetal veins 12

References 13


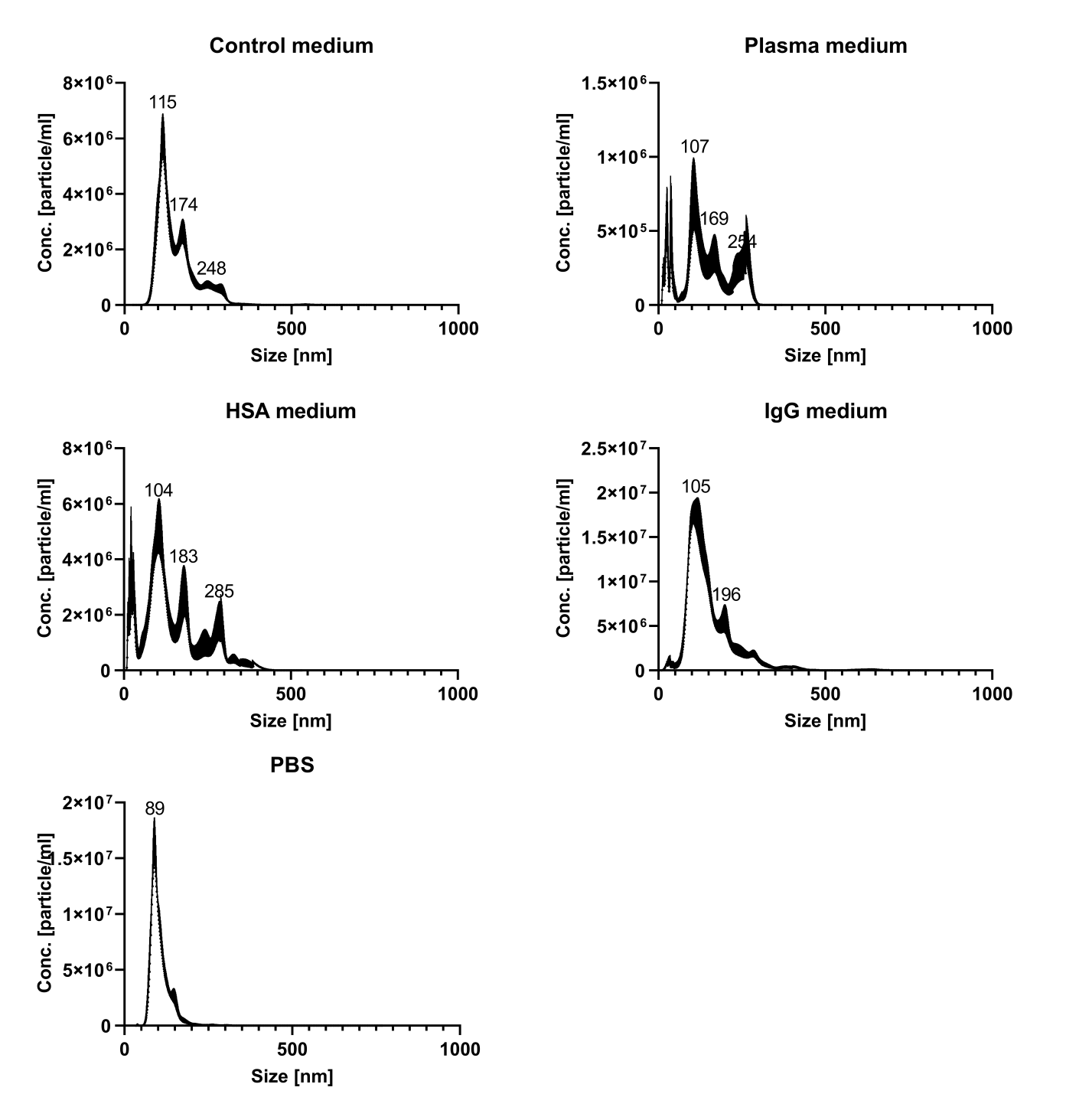


Supporting Figure 1 - Size distribution of 80 nm polystyrene particles. Particles (400ng/ml) in control-, plasma-, HSA-, IgG- medium compared to PBS measured by NTA. In all media the main PS fraction increased in size compared to the particle size in PBS.







**Supporting Figure 2 - Electron microscopic images.** Tissue was perfused either with PS in plasma medium (A) or with plain plasma medium (B). A) PS particles delineated as white circled structures (open arrow) were arranged within organelles (phagosomes, lysosomes or autophagolysosomes, filled arrow).^1,2^ B) In contrast, organelles (white arrow) of the control perfusion showed a heterogeneous pattern of incorporated vesicles.


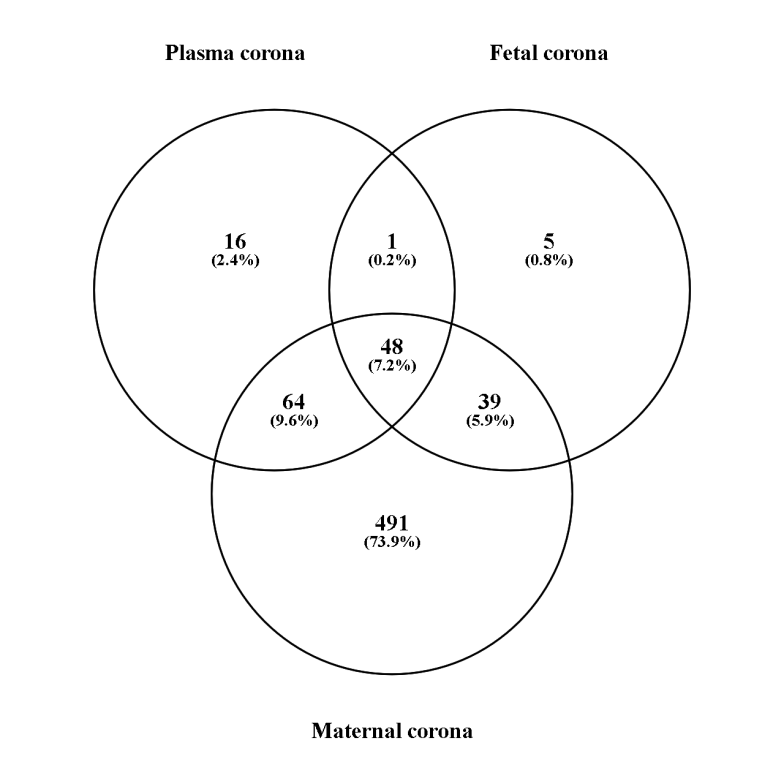


Supporting Figure 3 – Venn Diagramm. Qualitative protein distribution of proteins that were present in all 4 experiments in the respective group.^3^


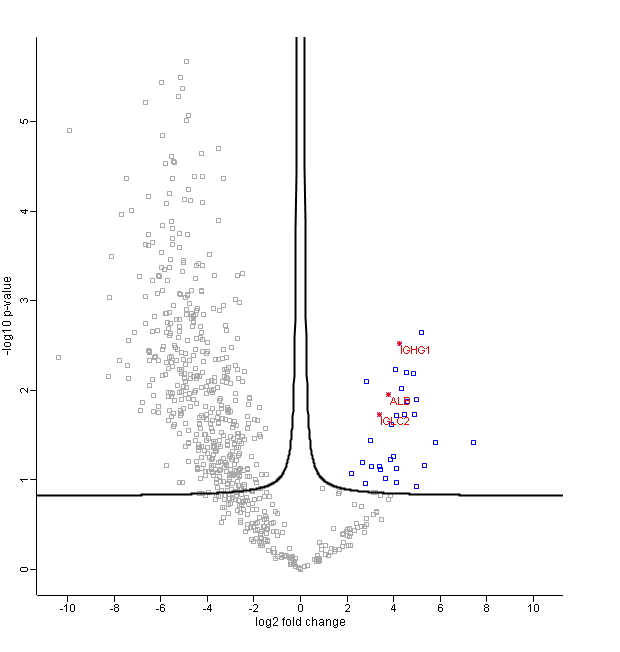


Supporting Figure 4 – Volcano plot. Comparison of particles isolated from fetal and maternal circulation after 360 min perfusion. Blue squares represent significanlty enriched corona proteins in particles from fetal circulation. Red squares show proteins chosen for further experiments. Supporting Table 5 summarizes gene names, normalized intensities, p-values and q-values of the volcanoplot.


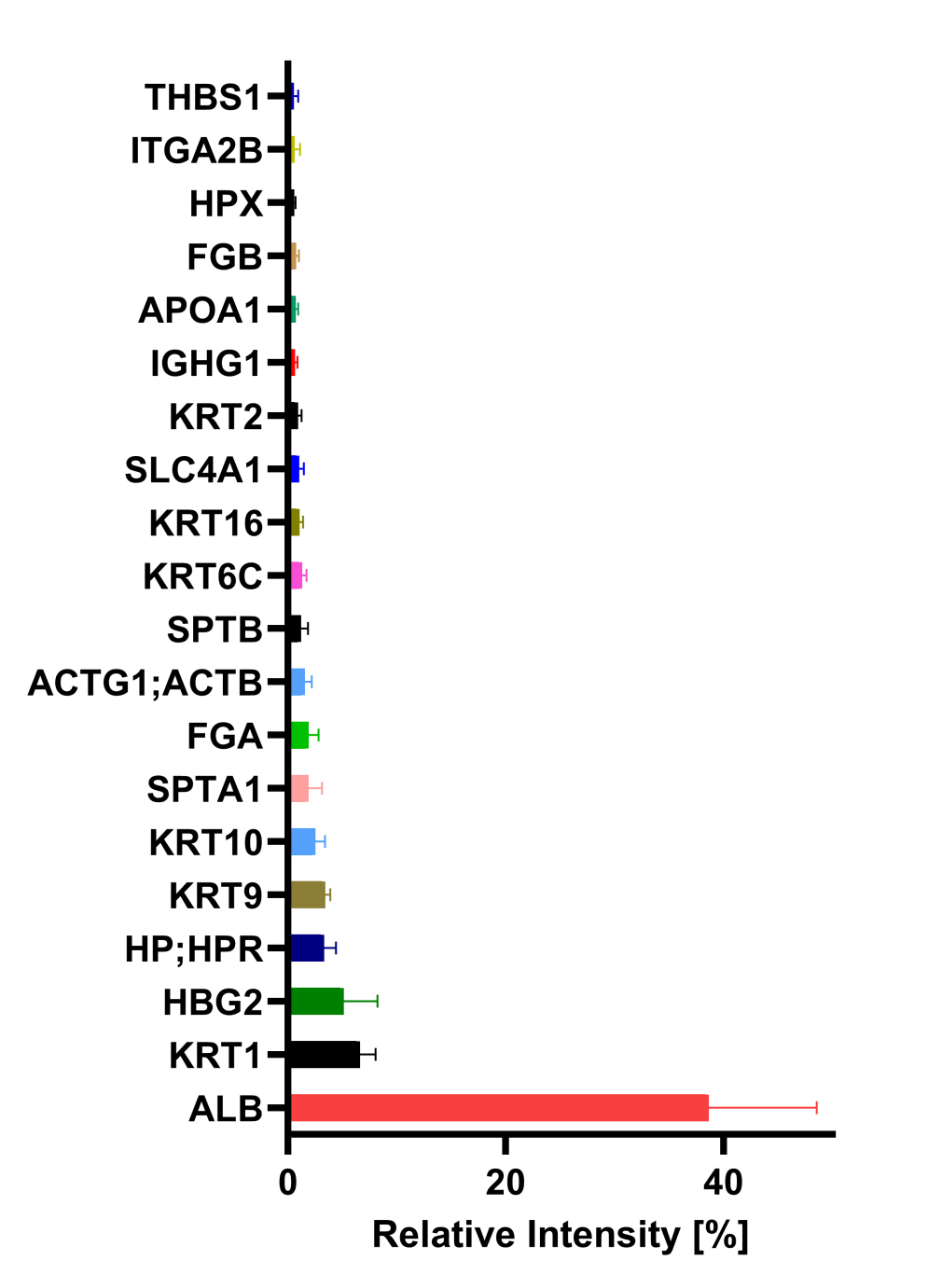


Supporting Figure 5 - Relative intensity of isolated fetal corona proteins. Proteins are presented with their respective gene name. Displayed proteins were significantly enriched in the fetal corona. Protein intensities are normalized to total protein intensity of the respective LC-MS/MS run. (Data are presented as mean ±SEM).

Supporting Table 1 – Statistics maternal arteries. Results of significant main and interaction effects generated with the linear mixed model for maternal arteries

| **Effect** | **media** | **time** | **media** | **time** | **Estimate** | **Standard Error** | ***DF** | **t Value** | **Pr > \|t\| (p-value)** |
| --- | --- | --- | --- | --- | --- | --- | --- | --- | --- |
| time | . | 15 | . | 30 | 3.5461 | 0.2210 | 34.6 | 16.05 | <.0001 |
| time | . | 15 | . | 45 | 6.2537 | 0.3139 | 53.9 | 19.93 | <.0001 |
| time | . | 15 | . | 60 | 8.2225 | 0.3769 | 34.2 | 21.81 | <.0001 |
| time | . | 15 | . | 90 | 10.8491 | 0.4545 | 21.9 | 23.87 | <.0001 |
| time | . | 15 | . | 120 | 12.4843 | 0.5291 | 18.6 | 23.60 | <.0001 |
| time | . | 15 | . | 180 | 14.4136 | 0.6210 | 15.9 | 23.21 | <.0001 |
| time | . | 15 | . | 240 | 15.2457 | 0.6804 | 15.2 | 22.41 | <.0001 |
| time | . | 15 | . | 300 | 15.7864 | 0.7194 | 14.9 | 21.94 | <.0001 |
| time | . | 15 | . | 360 | 16.2939 | 0.7859 | 14.4 | 20.73 | <.0001 |
| media*time | Control | 30 | Plasma | 30 | -8.3960 | 3.4178 | 20.8 | -2.46 | 0.0229 |
| media*time | Control | 45 | Plasma | 45 | -10.0112 | 3.4904 | 20.9 | -2.87 | 0.0092 |
| media*time | Control | 60 | Plasma | 60 | -9.1620 | 3.2937 | 21.3 | -2.78 | 0.0111 |
| media*time | Control | 90 | Plasma | 90 | -9.4120 | 3.0555 | 21.3 | -3.08 | 0.0056 |
| media*time | Control | 120 | Plasma | 120 | -8.7840 | 2.8873 | 20.8 | -3.04 | 0.0062 |
| media*time | Control | 180 | Plasma | 180 | -8.2680 | 2.6970 | 19 | -3.07 | 0.0064 |
| media*time | Plasma | 180 | HSA | 180 | 5.7025 | 2.6970 | 19 | 2.11 | 0.0479 |
| media*time | Control | 240 | Plasma | 240 | -7.4040 | 2.6071 | 17.7 | -2.84 | 0.0110 |
| media*time | Plasma | 240 | HSA | 240 | 5.5188 | 2.6071 | 17.7 | 2.12 | 0.0487 |
| media*time | Plasma | 240 | IgG | 240 | 6.4340 | 3.0104 | 17.7 | 2.14 | 0.0468 |
| media*time | Control | 300 | Plasma | 300 | -7.7060 | 2.5588 | 16.9 | -3.01 | 0.0079 |
| media*time | Plasma | 300 | HSA | 300 | 6.3051 | 2.5588 | 16.9 | 2.46 | 0.0248 |
| media*time | Plasma | 300 | IgG | 300 | 7.3647 | 2.9546 | 16.9 | 2.49 | 0.0234 |
| media*time | Control | 360 | Plasma | 360 | -7.6220 | 2.4680 | 15.2 | -3.09 | 0.0074 |
| media*time | Plasma | 360 | HSA | 360 | 6.4564 | 2.4680 | 15.2 | 2.62 | 0.0193 |
| media*time | Plasma | 360 | IgG | 360 | 7.7193 | 2.8498 | 15.2 | 2.71 | 0.0161 |

*DF…Degrees of Freedom

Supporting Table 2 – Statistics fetal arteries. Results of significant main and interaction effects generated with the linear mixed model for fetal arteries

| **Effect** | **media** | **time** | **media** | **time** | **Estimate** | **Standard Error** | ***DF** | **t Value** | **Pr > \|t\| (p-value)** |
| --- | --- | --- | --- | --- | --- | --- | --- | --- | --- |
| media | Control | . | HSA | . | -2.3110 | 0.8551 | 12.9 | -2.70 | 0.0182 |
| media | HSA | . | IgG | . | 3.1063 | 0.9873 | 12.9 | 3.15 | 0.0078 |
| time | . | 15 | . | 30 | -1.1491 | 0.1406 | 5.13 | -8.17 | 0.0004 |
| time | . | 15 | . | 45 | -2.2948 | 0.1927 | 5.29 | -11.91 | <.0001 |
| time | . | 15 | . | 60 | -3.2473 | 0.2286 | 10.6 | -14.20 | <.0001 |
| time | . | 15 | . | 90 | -4.5825 | 0.2859 | 16.1 | -16.03 | <.0001 |
| time | . | 15 | . | 120 | -5.5052 | 0.2147 | 51 | -25.64 | <.0001 |
| time | . | 15 | . | 180 | -6.5257 | 0.2447 | 55.2 | -26.66 | <.0001 |
| time | . | 15 | . | 240 | -6.8956 | 0.2380 | 39.7 | -28.97 | <.0001 |
| time | . | 15 | . | 300 | -6.9953 | 0.2401 | 27.4 | -29.14 | <.0001 |
| time | . | 15 | . | 360 | -7.0345 | 0.2464 | 23.8 | -28.55 | <.0001 |
| media*time | Control | 120 | HSA | 120 | -2.0021 | 0.8903 | 12.6 | -2.25 | 0.0431 |
| media*time | HSA | 120 | IgG | 120 | 2.8194 | 1.0280 | 12.6 | 2.74 | 0.0172 |
| media*time | Control | 180 | Plasma | 180 | -2.1300 | 0.9285 | 15 | -2.29 | 0.0367 |
| media*time | Control | 180 | HSA | 180 | -3.4569 | 0.9285 | 15 | -3.72 | 0.0020 |
| media*time | Plasma | 180 | IgG | 180 | 2.9980 | 1.0722 | 15 | 2.80 | 0.0136 |
| media*time | HSA | 180 | IgG | 180 | 4.3249 | 1.0722 | 15 | 4.03 | 0.0011 |
| media*time | Control | 240 | Plasma | 240 | -2.7940 | 0.8363 | 16.8 | -3.34 | 0.0039 |
| media*time | Control | 240 | HSA | 240 | -4.5348 | 0.8363 | 16.8 | -5.42 | <.0001 |
| media*time | Plasma | 240 | IgG | 240 | 3.6767 | 0.9657 | 16.8 | 3.81 | 0.0014 |
| media*time | HSA | 240 | IgG | 240 | 5.4175 | 0.9657 | 16.8 | 5.61 | <.0001 |
| media*time | Control | 300 | Plasma | 300 | -3.2160 | 0.7867 | 18.2 | -4.09 | 0.0007 |
| media*time | Control | 300 | HSA | 300 | -4.8992 | 0.7867 | 18.2 | -6.23 | <.0001 |
| media*time | Plasma | 300 | HSA | 300 | -1.6832 | 0.7867 | 18.2 | -2.14 | 0.0461 |
| media*time | Plasma | 300 | IgG | 300 | 4.2140 | 0.9084 | 18.2 | 4.64 | 0.0002 |
| media*time | HSA | 300 | IgG | 300 | 5.8972 | 0.9084 | 18.2 | 6.49 | <.0001 |
| media*time | Control | 360 | Plasma | 360 | -3.4500 | 0.7642 | 18.4 | -4.51 | 0.0003 |
| media*time | Control | 360 | HSA | 360 | -5.2059 | 0.7642 | 18.4 | -6.81 | <.0001 |
| media*time | Plasma | 360 | HSA | 360 | -1.7559 | 0.7642 | 18.4 | -2.30 | 0.0335 |
| media*time | Plasma | 360 | IgG | 360 | 4.6000 | 0.8825 | 18.4 | 5.21 | <.0001 |
| media*time | HSA | 360 | IgG | 360 | 6.3559 | 0.8825 | 18.4 | 7.20 | <.0001 |

*DF…Degrees of Freedom

Supporting Table 3 – Statistics maternal veins. Results of significant main and interaction effects generated with the linear mixed model for maternal veins

| **Effect** | **media** | **time** | **media** | **time** | **Estimate** | **Standard Error** | ***DF** | **t Value** | **Pr > \|t\| (p-value)** |
| --- | --- | --- | --- | --- | --- | --- | --- | --- | --- |
| media | Control | . | Plasma | . | -8.6376 | 2.7514 | 18.4 | -3.14 | 0.0056 |
| time | . | 15 | . | 60 | 1.1741 | 0.4182 | 21.1 | 2.81 | 0.0105 |
| time | . | 15 | . | 90 | 2.4514 | 0.5194 | 17 | 4.72 | 0.0002 |
| time | . | 15 | . | 120 | 3.1531 | 0.5724 | 17.1 | 5.51 | <.0001 |
| time | . | 15 | . | 180 | 4.4136 | 0.6316 | 16.2 | 6.99 | <.0001 |
| time | . | 15 | . | 240 | 4.9336 | 0.6836 | 15.7 | 7.22 | <.0001 |
| time | . | 15 | . | 300 | 5.4460 | 0.7333 | 14.8 | 7.43 | <.0001 |
| time | . | 15 | . | 360 | 5.7935 | 0.7498 | 14.3 | 7.73 | <.0001 |

*DF…Degrees of Freedom

Supporting Table 4 – Statistics fetal veins. Significant main effects generated with the linear mixed model for fetal veins

| **Effect** | **media** | **time** | **media** | **time** | **Estimate** | **Standard Error** | ***DF** | **t Value** | **Pr > \|t\| (p-value)** |
| --- | --- | --- | --- | --- | --- | --- | --- | --- | --- |
| time | . | 15 | . | 45 | 0.7264 | 0.2255 | 22.3 | 3.22 | 0.0039 |
| time | . | 15 | . | 60 | 1.0611 | 0.2743 | 19.5 | 3.87 | 0.0010 |
| time | . | 15 | . | 90 | 1.5357 | 0.3223 | 12.7 | 4.77 | 0.0004 |
| time | . | 15 | . | 120 | 1.7755 | 0.3576 | 12.2 | 4.96 | 0.0003 |
| time | . | 15 | . | 180 | 2.0643 | 0.3827 | 10.2 | 5.39 | 0.0003 |
| time | . | 15 | . | 240 | 2.1532 | 0.4052 | 12.4 | 5.31 | 0.0002 |
| time | . | 15 | . | 300 | 2.1725 | 0.4231 | 10.9 | 5.14 | 0.0003 |
| time | . | 15 | . | 360 | 2.1995 | 0.4361 | 11 | 5.04 | 0.0004 |
| media | Control | . | Plasma | . | -2.9892 | 1.0911 | 17 | -2.74 | 0.0140 |
| media | Control | . | HSA | . | -4.6954 | 1.0911 | 17 | -4.30 | 0.0005 |
| media | Plasma | . | IgG | . | 3.0710 | 1.2599 | 17 | 2.44 | 0.0261 |
| media | HSA | . | IgG | . | 4.7773 | 1.2599 | 17 | 3.79 | 0.0015 |

*DF…Degrees of Freedom

**References**

(1) Mühlfeld, C.; Rothen-Rutishauser, B.; Vanhecke, D.; Blank, F.; Gehr, P.; Ochs, M. Visualization and Quantitative Analysis of Nanoparticles in the Respiratory Tract by Transmission Electron Microscopy. *Part. Fibre Toxicol.* **2007**, *4* (February). https://doi.org/10.1186/1743-8977-4-11.

(2) Firdessa, R.; Oelschlaeger, T. A.; Moll, H. Identification of Multiple Cellular Uptake Pathways of Polystyrene Nanoparticles and Factors Affecting the Uptake: Relevance for Drug Delivery Systems. *Eur. J. Cell Biol.* **2014**, *93* (8–9), 323–337. https://doi.org/10.1016/j.ejcb.2014.08.001.

(3) Oliveros, J.C. (2007-2015) Venny. An interactive tool for comparing lists with Venn´s diagrams. https://bioinfogp.cnb.csic.es/tools/venny/index.html
